# The Role of Molecular and Inflammatory Indicators in the Assessment of Cognitive Dysfunction in a Mouse Model of Diabetes

**DOI:** 10.3390/ijms22083878

**Published:** 2021-04-09

**Authors:** Iwona Piatkowska-Chmiel, Mariola Herbet, Monika Gawronska-Grzywacz, Marta Ostrowska-Lesko, Jaroslaw Dudka

**Affiliations:** Department of Toxicology, Faculty of Pharmacy, Medical University of Lublin, 20-090 Lublin, Poland; mariola.herbet@umlub.pl (M.H.); monika.gawronska-grzywacz@umlub.pl (M.G.-G.); marta.ostrowska@umlub.pl (M.O.-L.); jaroslaw.dudka@umlub.pl (J.D.)

**Keywords:** diabetes, cognitive impairment, inflammatory markers, genetic markers, hippocampus

## Abstract

The brain is the most vulnerable organ to glucose fluctuations, as well as inflammation. Considering that cognitive impairment might occur at the early stage of diabetes, it is very important to identify key markers of early neuronal dysfunction. Our overall goal was to identify neuroinflammatory and molecular indicators of early cognitive impairment in diabetic mice. To confirm cognitive impairment in diabetic mice, series of behavioral tests were conducted. The markers related to cognitive decline were classified into the following two groups: Neuroinflammatory markers: IL-1*β*, IL-6, tumor necrosis factor-*α* (TNF-*α*) and genetic markers (*Bdnf*, *Arc*, *Egr1*) which were estimated in brain regions. Our studies showed a strong association between hyperglycemia, hyperinsulinemia, neuroinflammation, and cognitive dysfunction in T2DM mice model. Cognitive impairment recorded in diabetes mice were associated not only with increased levels of cytokines but also decreased *Arc* and *Egr1* mRNA expression level in brain regions associated with learning process and memory formation. The results of our research show that these indicators may be useful to test new forms of treatment of early cognitive dysfunction associated not only with diabetes but other diseases manifesting this type of disorders. The significant changes in *Arc* and *Egr1* gene expression in early stage diabetes create opportunities it possible to use them to track the progression of CNS dysfunction and also to differential disease diagnosis running with cognitive impairment.

## 1. Introduction

Diabetes (DM, diabetes mellitus) is a complex metabolic disorder which is characterized by hyperglycemia due to insulin insufficiency and/or insulin dysfunction. Currently, the number of patients with DM is increasing worldwide at an alarming rate. International Diabetes Federation and World Health Organization the observes increasing number of diabetes in the world and estimates that this trend will also continue in the future [1]. WHO estimates that diabetes will be the 7th most common cause of death in the world in 2030 (http://www.who.int/mediacentre/factsheets/fs312/en/ accessed on 8 June 2020). Diabetes is a chronic disease which may lead to the development of numerous complications [2,3,4,5,6,7,8,9]. One of the most dangerous consequences of chronic hyperglycemia are problems in function of central nervous system (CNS) which are referred as “diabetic encephalopathy” and is characterized by cognitive impairment and motor dysfunctions [10].

The brain is the most vulnerable organ to glucose fluctuations. Even slight changes in the level of glucose can lead to permanent neuronal damage and impairment of cognitive functions [11,12,13]. The results of neuropsychological tests in people with diabetes (especially in type 2) have shown mild or moderate dysfunction of cognitive activity [10]. The severity of above disorders were often correlated with serum level of glucose, duration of chronic hyperglycemia, and blood glucose fluctuations over a daily period [10]. The most prone areas of the brain affected by hypoglycemia are the hippocampus and prefrontal cortex. These areas of brain play important roles in forming and processing memories. As shown by Bree et al. [14], severe hypoglycemic episodes can lead to extensive neural damages in sensitive regions of hippocampus and cerebral cortex. The chronic hyperglycemia may also stimulate the production of pro-inflammatory cytokines like as IL 1, which further enhances glucotoxicity [15]. Nowadays, type 2 diabetes (T2DM) is mentioned as an important risk factor for dementia development. Patients with type 2 diabetes are about 50% more likely to develop dementia compared to the people without diabetes [16].

Some scientists started referring to Alzheimer’s Disease (AD) as type 3 diabetes to emphasize the potential endocrine links between these diseases [16,17]. Numerous literature reports indicate a close relationship between insulin resistance (IR) or hyperinsulinemia and cognitive, psychomotor impairment in patients with diabetes [18,19,20,21,22,23].

Recent research shows hyperinsulinemia being a form of response to hyperglycemia in type 2 diabetes may have a significant impact on its neuronal signaling. In the central nervous system, insulin plays key roles in learning and memorizing by the directing the secretion and reuptake of neurotransmitters. The decreased responsiveness of brain cells to insulin in regions such as hippocampus and cortex can lead to the disorder of synthesis of neurotransmitters and neuronal plasticity, which leads to cognitive impairments [22,23,24,25,26].

Additionally, brain insulin resistance increases the risk of hyperphosphorylation of the *tau* protein in the hippocampus and accumulations of beta-amyloid in the brain of diabetic patients, which may lead to neuronal dysfunction and cognitive deficits [20,27,28,29].

The cognitive decline observed in diabetes patients may also be associated with inflammatory changes in the brain. Inflammation, together with insulin resistance, is increased by expression of several pro-inflammatory cytokines such as interleukin IL-1, IL-6, and tumor necrosis factor (TNF-*α*) [27]. The activation and secretions of numerous pro-inflammatory mediators may also contribute to changes in multiple neurotransmitters synthesis such as serotonin, dopamine and also the increased blood–brain barrier (BBB) permeability and vulnerability of vessels as well as neuron damage and their premature death [30]. Experimental research show that release of pro-inflammatory cytokines such as Interleukin 1*β*, Interleukin 6, and TNF-*α* in diabetes may cause neuronal death and accelerate neurodegeneration characteristic for AD [31]. The elevated levels of proinflammatory cytokines in the brain of mice correlated with results of behavioral tests. Marioni et al. [32] confirmed the association of inflammation with the cognitive disorders in patients with diabetes. The levels of inflammation markers, like as IL-6 and TNF-*α*, were correlated with weaker cognitive functions of patients. Gorska-Ciebiada et al. [33] also correlated higher levels of inflammation markers (CRP, IL-6, and TNF-*α*) in diabetic patients with mild cognitive impairment.

Some studies show that TNF-*α* stimulates synthesis of other inflammatory mediators and generally worsens stroke patient’s condition [34,35]. TNF-*α* plays an essential role, in creating a linkage among insulin resistance [36].

Another cause of cognitive problems in diabetes patients may also be oxidative stress. The oxidative stress may play a key role in the late complications of diabetes through impaired neuronal insulin signaling, activation of advanced glycation end products (AGE/RAGE), polyol and protein kinase C (PKC) pathways, leading to increased brain inflammation and neurodegeneration [37]. The glycation end products (AGEs) can play a key role in formation and accumulation of neurotoxic β-amyloid in the brain not only in AD patients but also in diabetics [38,39].

Nowadays, much attention is paid to finding genetic biomarkers of early cognitive decline. The recent studies suggested that expression of *Bdnf*, *Arc*, *Egr1* genes may be related to cognitive functions. Literature data shows that, control of the above-mentioned genes expression is required to control neuronal plasticity, memory consolidation, and motor skill learning [40,41,42,43]. Various studies shown possible connections between the above-mentioned genes expression in the hippocampus and/or cerebral cortex and cognitive impairment in progress of diabetes, dementia, depression, schizophrenia, and Alzheimer’s disease (AD) [40,41,42,43]. Previous research indicates that one of the key factors predisposing to brain dysfunction in diabetes is chronic hyperglycemia. The results of experimental research indicate a relationship between chronic hyperglycemia in cell lines and/or brain neurons of diabetic animals and down-regulation of *Egr1* and *Bdnf* expression [44,45,46,47]. Furthermore, experimental studies proved that overexpression of some of these genes in the hippocampus allows for preservation of cognitive function and decreases brain lesion size in animals [40,48].

Given the number of factors that determine cognitive impairment in diabetes, it is very important to identify key markers of early neuronal dysfunction. Our overall goal was to identify neuroinflammatory and molecular indicators of early cognitive impairment in a diabetic mice model. In our study, we reveal behavioral, and molecular changes in areas of the brain related to learning and long-term memory formation that were noted early in diabetes in mice. The results of our research may be useful to test new forms of treatments of cognitive dysfunction associated not only with diabetes but other diseases manifesting this type of disorders. Moreover, significant changes in *Arc* and *Egr1* memory gene expression in early stage diabetes create opportunities it possible to use them to track the progression of CNS dysfunction and also to differential disease diagnosis running with cognitive impairment.

## 2. Results

### 2.1. The Impact of Diabetes on the Spontaneous Locomotor and Exploratory Activity and the Memory and Learning Process of Mice

**Figure 1:**
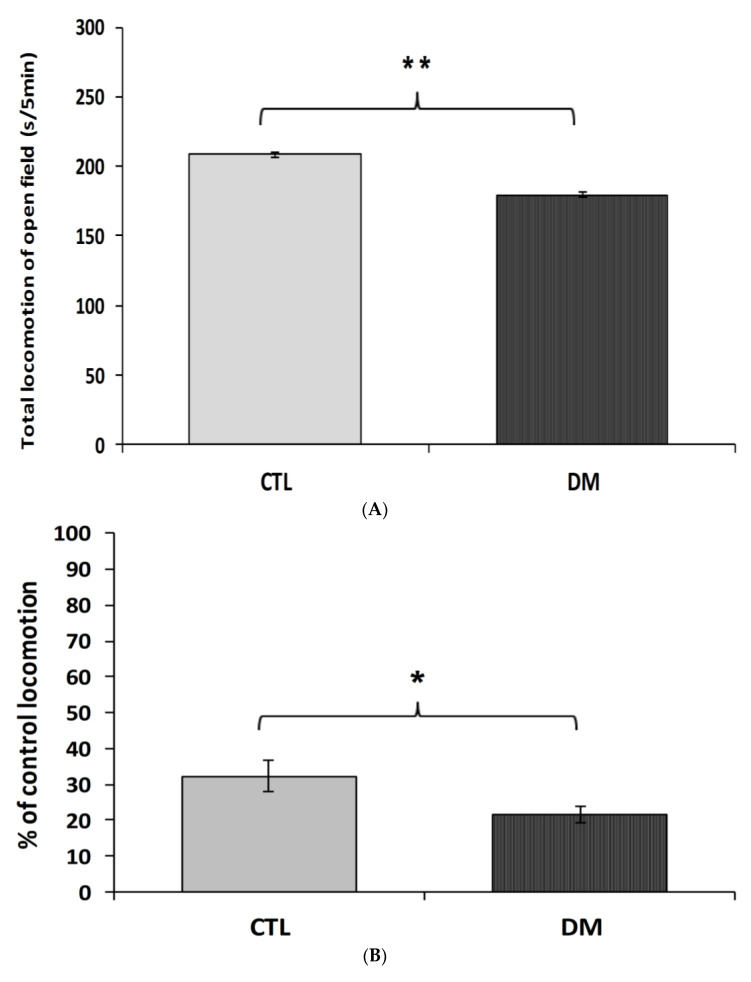

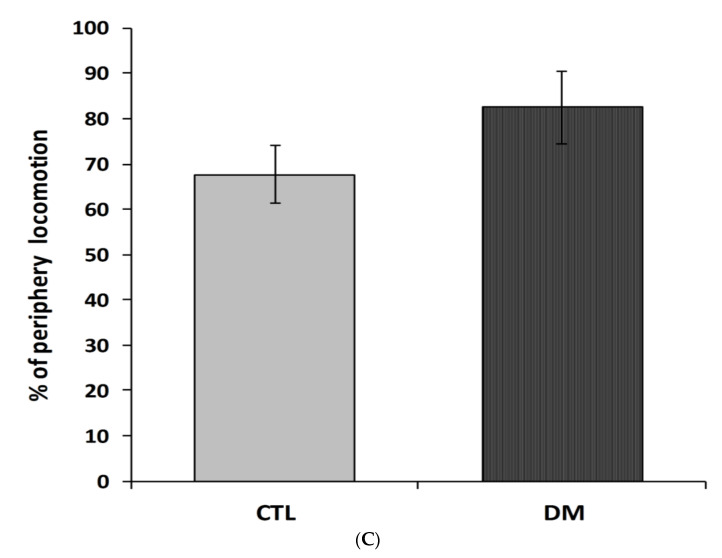
Spontaneous locomotor activity in diabetic mice. The locomotor activity was measured in terms of the squares number crossed (**A**), % time spent in the center zone (**B**), and % time spent in the periphery zone of the field (**C**). CTL: Control mice, DM: Diabetic mice. Each value represents mean ± SEM, *n* = 6. * *p* < 0.05; ** *p* < 0.01 vs. control.

To investigate the effects of diabetes on locomotor activity in mice, the open-field test was carried out. To measure animal activity we counted the number of exceeded squares by an individual and time spent in the central zone (the central four squares) and periphery of the field during 5 min of free exploration. As shown in Figure 1A diabetic mice were characterized by lower locomotor activity compared to the animals in control group (*p* < 0.01, t = 2.067, F = 3.241). Animals with diabetes spent significantly less time in central zone compared to animals in control group (Figure 1B, *p* < 0.05, t = 3.285, F = 3.895). In contrast, the locomotor activity of diabetic animals in the marginal zone of open-field was comparable to activity of animals in control group (Figure 1C, *p* > 0.05, t = 1.173, F = 1.247). Statistical analysis of the passive avoidance (PA) test results showed no significant (*p* > 0.05) difference in the latency to enter the dark compartment, during the conditioning trial, among experimental groups (data not shown).

**Figure 2:**
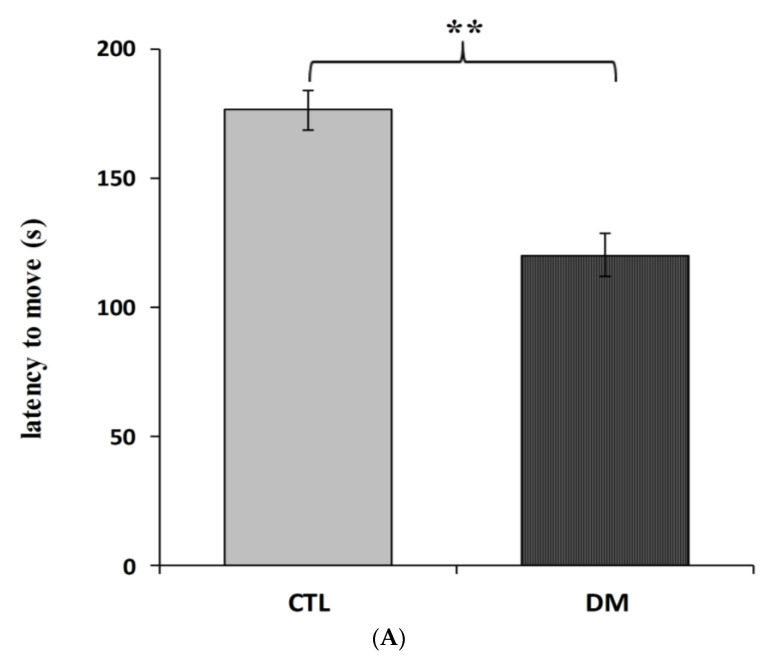

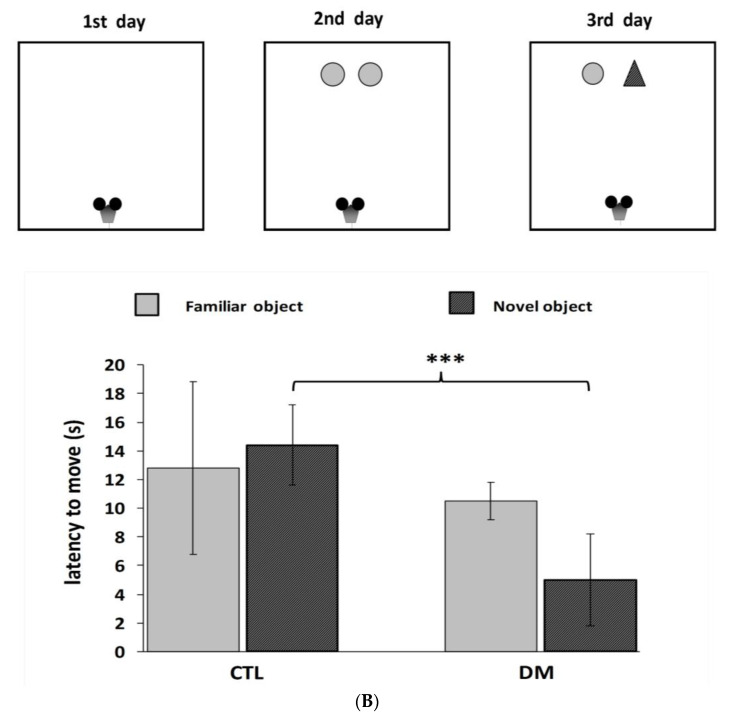
Influence of diabetes on learning and long-term memory process in mice. Passive avoidance (PA) test (**A**), “NOR-novel object recognition” test (**B**). Data are expressed as mean± SEM (*n* = 6). CTL: Control mice, DM: Diabetic mice. *** *p* < 0.001; ** *p* < 0.01 vs. control.

Furthermore, we recorded significant differences in the latency to enter the dark compartment between healthy and diabetic animals. Animals with induced diabetes showed a significantly shorter latency to enter the dark part of the apparatus 24 h after the application of an electrical stimulus compared with non-diabetic mice (Figure 2A; *p* < 0.01; t = 3.013 F = 5.386). Moreover, cognitive impairment in diabetic mice was also confirmed in “NOR—novel object recognition” test results. The performed statistical analysis showed that mice with induced diabetes showed a significant reduction in their interest in the presented objects, especially the new ones, compared to the animals in control group (Figure 2B, *p* < 0.001; t = 6.643, F = 1.315). Diabetic mice spent more time studying a familiar object (67% of time observation), while the % of mice that preferred the new facility was only 32%. In comparison, mice in the control group spent almost 60% of their time examining the new object. It is worth pointing out that during the familiarization phase, animals from all experimental groups spent a similar amount of time testing identical objects. The performed statistical analysis did not show any significant differences in the time of exploring the objects presented to the animals in both groups (*p* > 0.05).

Considering that spontaneous locomotion and the level of anxiety may affect the results of tasks related to learning and memory, therefore the results of these behavioral tests cannot indicate “pure” cognitive impairment in mice with diabetes.

### 2.2. Serum Level of Glucose (A), Insulin (B), HOMA-IR Index (C) in Mice

**Figure 3:**
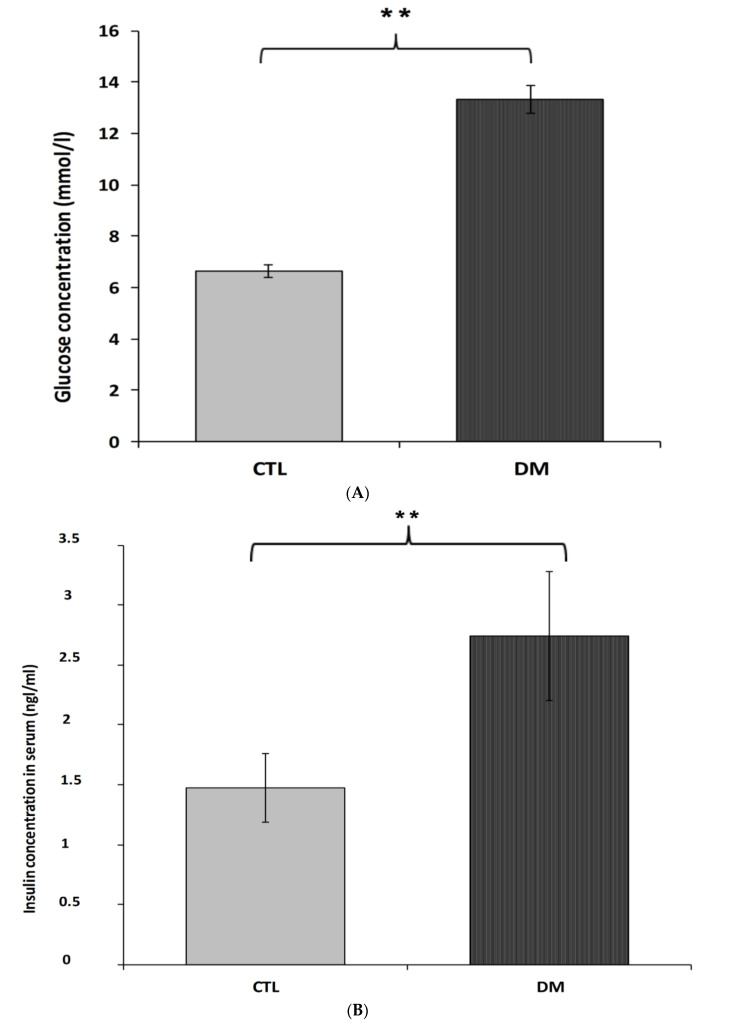

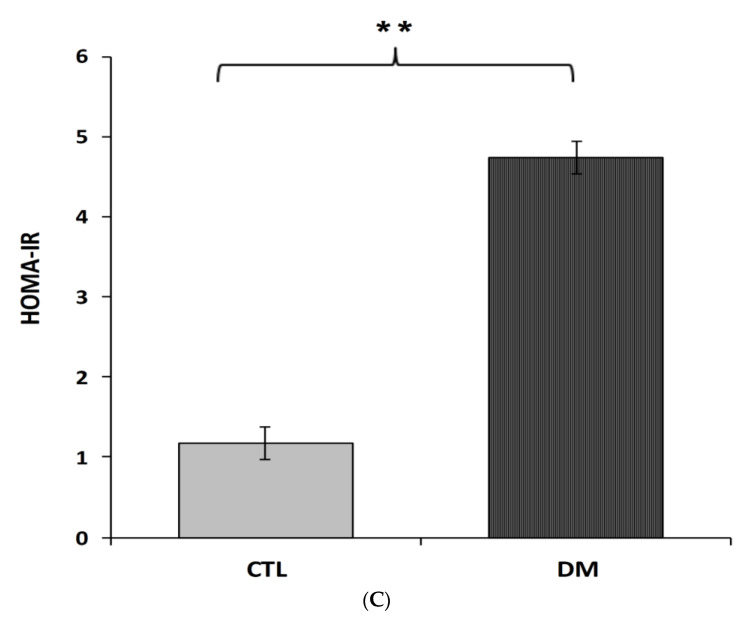
Level of glucose (**A**), insulin (**B**), HOMA-IR index (**C**) in the blood serum of diabetic mice. Data are expressed as mean ± SEM (*n* = 6). CTL: Control mice, DM: Diabetic mice. ** *p* < 0.01 vs. control.

Fasting serum concentrations of glucose and insulin in diabetic mice were significantly higher than animals in control group (Figure 3A,B). The average glucose level in the above-mentioned group was 13.3 mmol/L, which represented in 50% increase of this parameter compared to the control group (Figure 3A; *p* < 0.01, t = 3.175, F = 3.343). Similar changes were observed in insulin levels. Furthermore, nearly 50% increase of this hormone in the serum of diabetic animals compared to healthy animals (Figure 3B; *p* < 0.01, t = 2.088, F = 3.533) was recorded. Results obtained from this experiment indicated that the current model of type 2 diabetes mellitus exhibited higher HOMA-insulin resistance index (HOMA-IR) compared to control group mice (Figure 3C; *p* < 0.01).

### 2.3. Cytokine Level in Prefrontal Cortex of Mice

**Figure 4:**
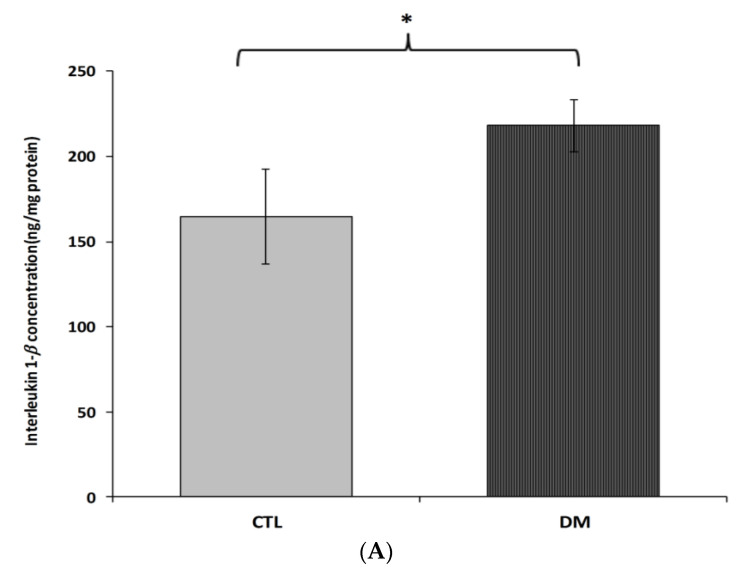

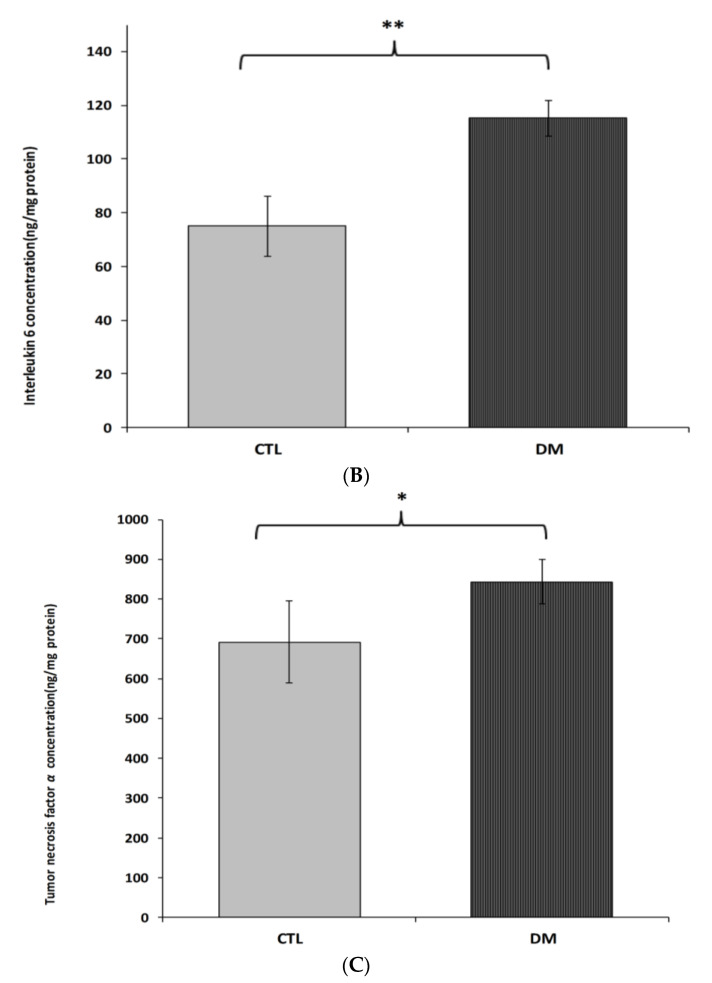
Levels of IL-1*β* (**A**), IL-6 (**B**) and TNF-*α* (**C**) in brain prefrontal cortex of diabetic mice. Data are expressed as mean ± SEM (*n* = 6). CTL: Control mice, DM: Diabetic mice; * *p* < 0.05, ** *p* < 0.01 vs. control.

Interleukin 1*β*, interleukin 6 and tumor necrosis factor α brain cortex levels in diabetic mice were significantly higher than in the control group (Figure 4A–C). Interleukin 1*β* levels were about 32% higher in diabetic mice than in healthy ones (Figure 4A; *p* < 0.05, t = 2.064, F = 3.543). Similar change was observed in interleukin 6 levels. We recorded nearly 35% increase in the level of this cytokine in the prefrontal cortex of diabetic mice compared to healthy animals (Figure 4B; *p* < 0.01, t = 3.230, F = 2.297). In the case of tumor necrosis factor *α*, an increase of around 20% in the prefrontal cortex of diabetic animals compared to the healthy adult mice was observed (Figure 4C; *p* < 0.05, t = 2.832, F = 7.543).

### 2.4. Insulin Level in Prefrontal Cortex of Type 2 Diabetes Mice

**Figure 5:**
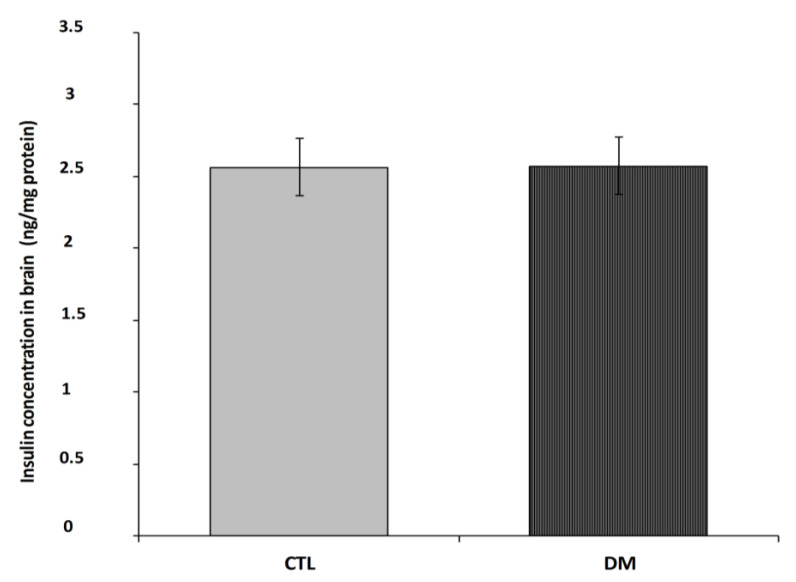
Insulin level in prefrontal cortex of diabetic mice. Data are expressed as mean ± SEM (*n* = 6). CTL: Control mice, DM: Diabetic mice.

Figure 5 shows insulin levels in prefrontal cortex in 2 subject groups; normal adult mice and diabetes mice. Brain insulin levels in the diabetic mice were close to values of non-diabetic group (Figure 5; *p* = 0.2731, t = 1.177, F = 1.600).

### 2.5. mRNA Expression Analysis in Hippocampus and Prefrontal Cortex of Mice

**Figure 6:**
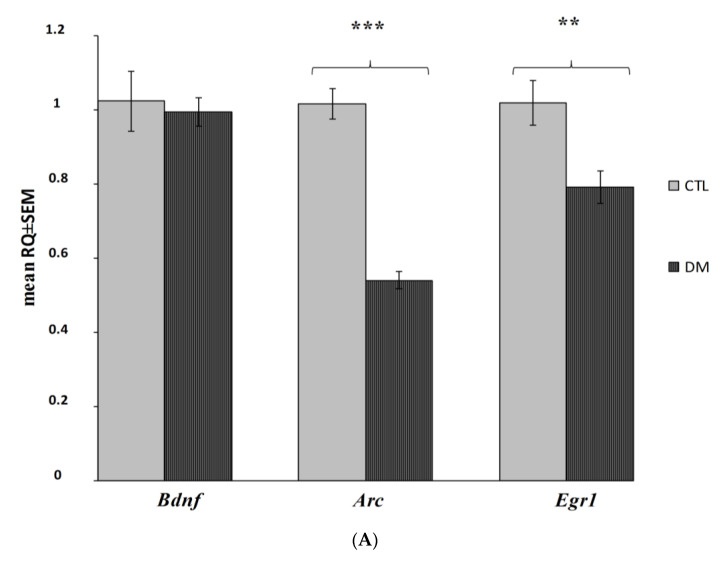

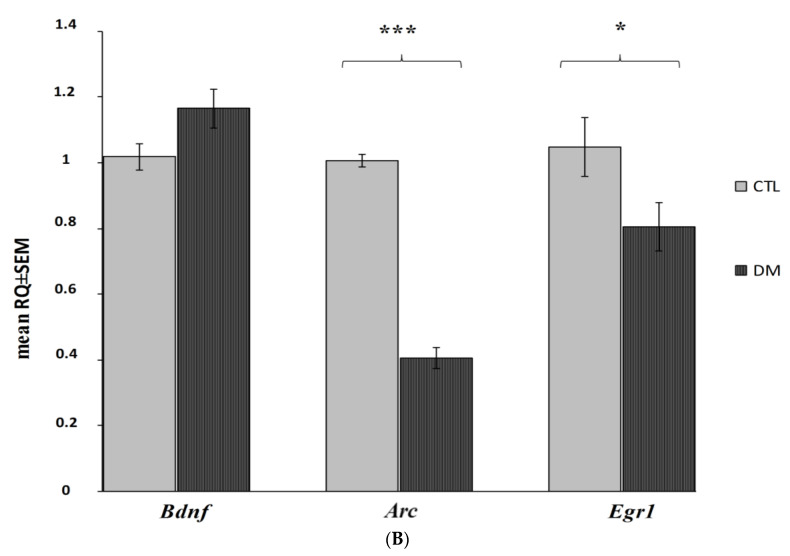
Changes in mRNA expression level in the prefrontal cerebral cortex and hippocampus in the analyzed in diabetic mice (*n* = 6 mice per group). Data presented as RQ ± SEM. *** *p* < 0.001; ** *p* < 0.01, * *p* < 0.05 versus control group prefrontal cortex (**A**), hippocampus (**B**).

The analysis of gene expression showed that the presented model of diabetes had a statistically significant impact on the decrease of *Arc* and *Egr1* mRNA expression levels in both prefrontal cerebral cortex and hippocampus (Figure 6A,B; *p* < 0.001; *p*  <  0.01; *p*  <  0.05). Our data indicated that *Arc* expression was significantly reduced in both the hippocampus and cerebral cortex, whereas *Egr1* was affected especially in the cortex and to a slightly lesser degree in the hippocampus. However, the diabetes did not significantly affect the *Bdnf* expression in the examined brain regions (Figure 6A,B; *p* > 0.05).

## 3. Discussion

It is well known that the metabolic and vascular disorders characteristic of diabetes very often lead to the development of numerous complications in the peripheral as in the central nervous system.

The analysis of the available literature shows that both prolonged hyperglycemia and insulin resistance as well as hyperinsulinemia affect the progression of this disease as well as related complications such as deficits of cognition and psychomotor function. The epidemiological studies show that those with diabetes have poorer cognitive performance, particularly in verbal memory and complex information processing [10].

The results of our studies showed a positive correlation between indicators of diabetes and cognitive dysfunction in diabetic mice. High fasting glucose and insulin levels and high HOMA-IR index didn’t only confirm effective induction of experimental type 2 diabetes but also made it possible to observe CNS dysfunction widely described in the literature [6,7,11,13,49]. Moreover, diabetic mice were characterized by lower locomotor and cognitive activity compared to the animals in control group.

As shown in research by Bree et al. [14], chronic hyperglycemic and hypoglycemic episodes can lead to extensive neurons damage in sensitive regions of hippocampus and cerebral cortex which plays an important role in forming and processing memories. Moreover, chronic hyperglycemia promotes increased permeability of the blood–brain barrier (BBB) and penetration of toxic substances to the CNS which of course promotes neuroinflammation and cognitive decline in DM patients [50,51,52].

Numerous literature reports indicate also a close relationship between hyperglikemia, insulin resistance (IR) and cognitive, psychomotor impairment in patients with diabetes [14,53,54,55]. Our results also confirm this relationship. Dysfunction of learning and memory process in animals were positively correlated with serum glucose and insulin level. We observed nearly 50% increase in level of this hormone in the serum of diabetic animals compared to animals in control group. More and more studies assigns PKC and MAP kinases participation in diabetic vascular complications in nervous system and related to it psychomotor impairments [56,57]. Hyperglycemia through modulating of PKC activity it may lead to damage neuronal cells by impairing vasodilation and increasing capillary thickening and endothelial hyperplasia, which diminish neuronal blood flow and oxygen availability for cells [58]. Additionally, hyperglycemia may reduces Na^+^K^+^ ATPase activity, which is essential for maintaining normal nerve membrane resting potential [59].

Moreover, hyperinsulinemia being a reaction to hyperglycemia in type 2 diabetes and may have a significant impact on its neuronal signaling. Experimental and clinical studies [20,23,52,55] indicate a significant role of insulin in synaptic plasticity of hippocampal cells and the cerebral cortex. Insulin in the brain, may function as a neuromodulator directing the secretion and reuptake of neurotransmitters, affecting learning and memory process [60]. Research Zhao et al. [61] showed that rats after training in a water maze had increased insulin mRNA levels in the hippocampus, as well as increased accumulation of insulin receptor proteins.

As already known, in animal models of diabetes of spatial learning deficits are paralleled by neurophysiological and structural changes in the brain. At the molecular level these impairments might involve changes in glutamate-receptor subtypes, in second-messenger systems in protein kinases and changes receptors sensitivity through on endogenous substances like as insulin [61,62].

Our research showed that despite confirmed peripheral hyperinsulinemia, and presence of cognitive impairment, we did not notice any significant changes in brain insulin level in diabetic animals vs. non-diabetic mice, which of course, does not exclude the possible changes in the transport of this peptide across the BBB.

Moreover, the lack of significant differences in insulin levels in the brains of non-diabetic group with diabetes could possibly dictated by insulin-degrading enzyme (IDE) activity in diabetes mouse. More and more research is focusing on the role IDE (insulin-degrading enzyme) in modulate insulin level, arising amyloid β (Aβ) and development of cognitive impairment characteristic for the type 2 diabetes mellitus (T2DM) and Alzheimer’s disease. Some studies have showed that IDE increased could mediate of progressive brain insulin deficiency and insulin resistance in AD [63,64,65]. With turn deficiency in IDE might lead to an accumulation of insulin in the brain (hyperinsulinemia), leading to insulin resistance and glucose intolerance [66]. This area thus requires further research.

Every year we receive new data that confirms the existence of a close relationship between levels of markers and mediators of inflammation of diabetes and its neurological complications [47,67,68]. There is a plethora of evidence describing the close relationship between production of pro-inflammatory cytokines such as interleukin IL-1, IL-6 and tumor necrosis factor (TNF)-*α*, and neuronal death and accelerate neurodegeneration process characteristic for AD [26,69,70,71].

The results of our work confirm this relationship. The cognitive disturbances were positively correlated with levels of pro-inflammatory cytokines such as Interleukin 1-*β*, interleukin 6 and tumor necrosis factor α in prefrontal cortex in the brain of diabetic mice. The onset of diabetes caused a higher brain level of inflammatory indicators as compared to the healthy control subjects. Specifically, IL-1*β* and IL-6 were significantly correlated with changes of cognition. Interleukin 1-β and interleukin 6 levels were higher about 32–35% and in the case of TNF, around 20% higher than in control group.

Research shows that in diabetes, the abnormally differentiated vascular endothelia cells and perivascular macrophages may show an exaggerated inflammatory response characterized by an in-creased secretion of pro-inflammatory cytokines, such as TNF-*α*, IL-1*β* and iNOS [72]. Some studies confirm that diabetes may accompanied also activation of glial cells is exaggerated, which leads to releases large amounts of inflammatory agents [70,73].

On the other hand chronic inflammation may leads to degeneration a specific neuronal populations and their premature dying as it happens in Alzheimer’s disease. A both increased production and impaired elimination the cell debris i.e., damaged organelles, and macromolecules may increase inflammation process in the CNS [74].

It has also been shown that the activation of a central inflammatory response and the accompanying level of the liberated cytokines to correlate with changes in multiple neurotransmitters metabolism such as serotonin, dopamine and glutamate. Moreover, cytokines, by activating the kynurenine pathway, may effectively limit synthesis of serotonin, but also participate in the generates neuroactive metabolites that can significantly influence the regulation of dopamine and glutamate [75,76].

This negative impact of inflammatory cytokines on neurotransmitter systems may leads to significant changes in motor activity and motivation as well as development of anxiety disorders. Our research results confirm this relationship. The diabetic mouse were characterized by lower locomotor activity and higher anxiety level compared to the animals in control group.

The analysis of the available studies indicates the essential role glycogen synthase kinase-3β in development cognitive impairment in diabetes [77,78].

Datusalia et al. [78] showed that the modulation of GSK-3β signaling is able significantly reduced neuroinflammation of diabetic rats by reduction in IL-6, TNF-*α*, COX-2, which also it was reflected in the neurotransmitters level the hippocampus and cortex.

A plethora of data demonstrates that neuroinflammation or altered hypothalamic–pituitary–adrenal axis functionality can affect genes expression which regulate synaptic function and are a critical factor responsible for memory consolidation [41,52,79]. Although, studies show that BDNF level may decrease in T2DM in response to hyperglycemia, we did not observe such a correlation in our research. *Bdnf* mRNA expression level in both prefrontal cerebral cortex and hippocampus remained at a similar level as in healthy animals [47,67].

The lack of a noticeable difference in the expression of Bdnf gene between the study groups may arise from early-stage of diabetes. Kauer-Sant’Anna M et al. [68] noticed that BDNF level appeared to be related to the illness duration and may remain unchanged during the early phase of the CNS disorders.

Krabbe et al. [47] also Passaro et al. [80] observed inverse relation of serum BDNF level to fasting glucose and duration of T2DM and cognitive function.

Given no differences in *Bdnf* mRNA expression level in the studied groups we ruled out the existence of disorders in BDNF signaling in the course of diabetes, although we know that they may have a negative effect on genes expression involved in synaptic plasticity, including Homer1a, *Arc* [47].

Numerous studies prove a strong correlation between neuronal activity and *Erg1* and *Arc* expression exists [40,41,42,81]. Numerous studies prove that *Erg1* and *Arc* genes are widely used as molecular indicators of synaptic plasticity in brain regions associated with learning and long-term memory formation [81,82,83,84,85,86].

Taking into consideration that brain gene expression is dynamically changed especially within the immediate-early genes (IEGs) group (such as *Egr-1* and *Arc*), our research focused mainly on assessing their expression and evaluating their potential usefulness as genetic indicators of early cognitive impairment in diabetes.

Our data indicated that *Arc* and *Erg1* genes expression is associated with nervous system function, and particularly neurotransmission, learning, and memory, and that significantly reduced expression in both brain regions was recorded. *Arc* is required for numerous learning and memory tasks, including memory consolidation, spatial learning and memory [87]. *Arc* expression was significantly reduced in both the hippocampus and cerebral cortex, whereas *Egr1* was affected especially in the cortex and to a lesser degree in the hippocampus. Given the importance of *Arc* and *Egr1* in memorizing, it is plausible that their reduced expression is the reason for the memory deficits observed in results of behavioral tests.

Animals with induced diabetes showed a significantly shorter latency to enter the dark part of the apparatus after 24 h after the application of an electrical stimulus compared with non-diabetic mice. Similarly, as *Egr1^−/−^* mouse in a test of object recognition showed a significant reduction in preference for presented novel object 24 h after training [84].

It should also be noted that reduced *Arc* and *Egr1* expression and related memory problems may also be a consequence of neuroinflammation. Expression of these two genes showed to be lowered in association with β-amyloid plaques in AD patients, animal models of AD and aged mice [85,88,89]. In our study it was proved that diabetic mice showed a significant reduction in their interest in the novel objects when compared to the animals in control group. Penrod et al. [87] proved that the reduction of *Arc* produced significant deficits in both object and social novelty preference tasks what is also in line with our observations and it pointed out that the induction of *Arc* is necessary for normal novelty behavior suggesting inter alia this gene as a regulator of detection of novelty [87]. In addition, recent study proved that mice deficient in EGR-1 (*Egr1^−/−^*) fed with high-fat diet failed to secrete sufficient insulin to clear glucose, which was associated with lower insulin content and the development of pancreatic islet failure [90]. It is known that early growth response gene 1 (*Egr1*) is implicated in the regulation of cell differentiation, proliferation, and apoptosis. The deficiency of EGR-1 might influence β-cell compensation in response to metabolic overload what [90]. These findings highlight the importance of this gene in the future studies focused on diabetes mellitus.

## 4. Materials and Methods

### 4.1. Chemicals

In the experiment were used: Streptozotocin (≥98% HPLC, Sigma-Aldrich, Munich, Germany) which was freshly prepared in citrate buffer (0.01 M, pH = 4.5); saline (aqua pro injection, Baxter, Lublin, Poland). Citric acid and sodium citrate were supplied by Biomed Company (Lublin, Poland).

### 4.2. Animals

The experiment was conducted on adult male mice CD-1 of 7 to 8 weeks old (output weight of 20–24 g). The mice were obtained from a licensed breeder (Center of Experimental Medicine, Medical University of Lublin, Poland) and were grown and housed in a pathogen-free facilities in accordance with the Regulation of the Minister of Agriculture and Rural Development of 14 December, 2016 (Journal of Laws item 2149). Mice were housed in groups of 4 per cage. These animals did not show any apparent in their behavioral abnormalities which could be evidence of congenital anomalies. Before and during the experiment, the animals were subjected to active and inquisitive observation by an experienced veterinarian (moving around the cage, grooming, eating, drinking, defecation and urination, and interacting with cage mates).

Additionally, every mouse was hands-on examination by the vet which allowed for assessment of hydration status, body condition, and the presence of abnormalities in the bones, genitals and abdomen. Body weight of mice were recorded weekly. All procedures were approved by the Local Ethics Committee on Animal Experimentation in Lublin (No. 43/2018).

### 4.3. Experimental Procedures

#### 4.3.1. Induction of Type 2 Diabetes Mellitus (DM) Mouse Model

The experiment was divided into two stages. In the first stage, male mice (CD-1) were randomly divided into 2 experimental groups: A control group (non-diabetic mice- CTL), diabetics mice (DM). Then in one of experimental groups was started the procedure of diabetes induction. During the first 4 weeks of the experiment, the animals were allowed to drink aqueous 20% fructose solution and then, for the next 5 days (1 × daily) mice were administrated intraperitoneal injection of freshly prepared solution of STZ (40 mg/kg *ip* body weight) in 0.01 M cold citrate buffer pH 4.5.

**Figure 7:**
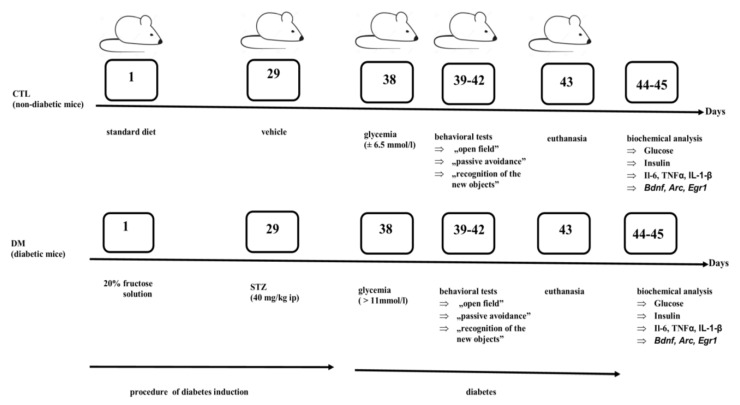
The experimental mouse model of type 2 diabetes mellitus (DM).

At the same time the control mice were administrated citrate buffer alone. Throughout the duration of the experiment all animals had free access to feed and fluids throughout the experiment. In the next stage, nine days after the first STZ injection, blood samples were collected from the tail vein of animals. To estimate the blood glucose level was used advanced Glucometer ACCU-CHEK (Roche, Mannheim, Germany). Animals with a fasted glucose level >11 mmol/L were qualified for the next stage and behavioral tests were carried out to evaluate the effect of diabetes on the cognitive functions of animals. Control animals had blood glucose level on average 6.5 mmol/L. The scheme of the experimental model is presented in Figure 7.

Diabetes induction started on the first day of the experiment. During the first 28 days of the experiment, the animals were allowed to drink aqueous 20% fructose solution or water and eat standard feed. Then, from the 29th day mice received an intraperitoneal (ip) injection of streptozotocin (STZ) or vehicle. After nine days after the first STZ injection, (on the 38th day of experiment), in animals was determined blood glucose level. Between 39–42 days, behavioral tests were performed. On the 43th day, the animals were sacrificed and biological material (blood samples and brains) was collected. Biochemical parameters were determined between 44 and 45 days.

#### 4.3.2. Procedures of Behavioral Tests

All behavioral tests were carried out under controlled environmental conditions, such as temperature, humidity, and light intensity (dim illumination). In order to avoid possible circadian modifications of the test results, all experiments were carried out between 9.00 a.m. and 11.00 a.m. In order to eliminate olfactory cues were systematically cleaned all apparatuses.

Behavioral tests that we used to assessment of the spontaneous locomotor and exploratory activity of rodents were: “Open field” test, while the “passive avoidance” and “recognition of new objects” tests were used to assessment the memory and learning process of mice.

In the “open field” test [91] the animal was placed in the central part of the field measuring 40 × 40 × 35 cm, made of natural wood. The floor of the field was divided into 16 equal squares. The assessment of locomotor activity was based on the number of squares exceeded by a given individual and time spent in the central zone (the central four squares) and periphery of the field during 5 min of free exploration.

The assessment of learning and long-term memory carried out with the use of passive avoidance (PA) test (24 h after the previous test) in which rodents learn to restrain their innate tendency, namely preferring a dark compartment rather than an illuminated.

This test was carried out in a special apparatus which consisted of a black dimly lit chamber (25 × 20 × 15 cm) and a white illuminated chamber (10 × 13 × 15 cm) [92]. Both parts of the apparatus were separated by a wall with a gate between them. The floor of the dark part of the apparatus was connected to a constant voltage power source.

The test was carried out in two stages. On the first day, animals were placed individually in the illuminated compartment and allowed to freely scour for 5 min. Then, after 15 min following habituation was started the first phase of the test. The mouse was placed again in the lighted part of the chamber and when the animal crossed to the black compartment, it was received a mild foot shock which was to be a negative learning spur. After completing this stage of the test, the animal learned that the moving to the dark compartment may have negative consequences. On the next day (24 h later), the same animals were put into the illuminated box and observed up to 180 s. If mice went into the dark part of the camera (aversive stimulus) during 180 s, this indicated a memory impairment. The longer the delay were in the animals’ transition to the dark part of the camera, the better their memory.

The procedure of “NOR-novel object recognition” test was carried out based on modifications of the Ennaceur and Delacour method [93]. This test is based on the tendency of rodents to discover novel objects.

The test was carried out in a wooden, white box with dimensions of 40 cm × 40 cm × 35 cm (height). Wooden cubes in the shape of a rectangular or quadrangular pyramid (4 cm × 4 cm × 6 cm) were used as objects. On the first day, all mice underwent an adaptation session during which they could freely explore the open field for 10 min. During the aforementioned process, the animals did not come into contact with any of the objects. Twenty-four hours after the taming test, the animals were again placed in the crate for 10 min, of which after the first 5 min were placed two identical objects (A + A) in the crate at an equal distance from each other, 9 cm from the walls of the crate (familiarization phase). All objects (blocks) were made of the same material (wood) and they had the same color (white) and size (weight, height, and width). Each mouse could freely examine both objects. After 24 h, the test phase began. Each mouse was placed in an empty chest for 5 min for adaptation, and then objects were introduced into the chest: One known and the other new (A + B). During the 5 min of observation, the time of exploring each object by mice was recorded.

The interaction with the object was defined as sniffing or touching with the nose (the mouse’s head was 2 cm away from the object and was directed towards it). Resisting, climbing on blocks were not considered as a form of exploration. After each test, the box and objects (blocks) were cleaned with a 10% ethanol solution to eliminate odor signals.

The [%] of the level of mouse preference for the new object was calculated as the time of interaction of the mouse with the new object/the sum of interaction time with the familiar and new object × 100.

Twenty-four hours after the behavioral tests (43rd day), mice were killed by decapitation while keeping animals for the last 4–6 h of fasting. After decapitation, 1 mL trunk blood was collected at the decapitation site. The verification of the type 2 diabetes mellitus (T2DM) model was based on determination of serum levels of glucose and insulin and estimation of insulin resistance using the HOMA-IR index. At the same time, the brains were removed followed by the isolation of the hippocampus and prefrontal cortex for determining TNF-*α*, IL-1*β*, IL-6 levels, and genes expression from each animals.

### 4.4. Quantitative Determination of Serum Levels of Glucose and Insulin and Estimation of Insulin Resistance Using the HOMA-IR Index

To obtain the serum, the blood were collected in tubes without any anticoagulant and allowed to clot. Blood samples were centrifuged at 1000× *g* for 10 min. Then, serum samples were collected into two clean Eppendorf’s tubes and stored at −20 °C until used for the ELISA assays. In the received serum was determined the levels of glucose and insulin. For the determination of insulin levels was used mouse enzyme-linked immunosorbent assay (ELISA) kit (Mercodia AB, Uppsala, Sweden). Mercodia Mouse Insulin ELISA is a solid phase two-site enzyme immunoassay based on the sandwich technique, in which two monoclonal antibodies are directed against separate antigenic determinants on the insulin molecule.

Insulin in the sample reacts with specific anti-insulin antibodies bound to wells of the microplate supplied by the manufacturer of kit. After incubation unbound antigen was washed away and were added peroxidase-conjugated anti-insulin antibodies in the solution. After 2 h incubation at room temperature on a shaker the wells were washed few times, after that substrate solution was added to each well. The added substrate reacted with the enzyme–antibody–insulin complex to produce measurable signal. The intensity of signal obtained at 450 nm was directly proportional to the insulin concentration of the present specimen. The serum insulin concentration was determined by comparison with the standard curve. Results were expressed as ng/mL and the limit detection was ≤0.2 ng/mL. The procedure was carried out following manufacturer instruction.

The serum glucose level was determined immediately using a glucose diagnostic kit (Liquick-Cor Glucose, Cormay, Warszawa, Poland). This kit is for the quantitative, enzymatic determination of glucose in serum. This method is based on enzymatic reaction, in which glucose is oxidized to gluconic acid and hydrogen peroxide by glucose oxidase. Then hydrogen peroxide reacts with 4-aminoantipyrine in the presence of peroxidase to form a red colored product. The intensity of the red color was measured at 500 nm after 5 min. incubation (samples or standard with the 1-Glucose reagent) at 37 °C.

$$\mathrm{Calculation}\;\mathrm{of}\;\mathrm{glucose}\;\mathrm{concentration}\;=\frac{\mathrm{absorbance}\;\mathrm{of}\;\mathrm{sample}}{\mathrm{absorbance}\;\mathrm{of}\;\mathrm{standard}}\times \mathrm{standard}\;\mathrm{concentration}\;\mathrm{of}\;\mathrm{glucose}\;\left(5.5\;\mathrm{mmol}/L\right).$$

Results were expressed as mmol/L. The procedure was carried out following manufacturer instructions.

Insulin resistance was estimated using the formula: HOMA-IR  =  fasting glucose (mmol/L) × fasting insulin (mU/L)/22.5 [94].

### 4.5. Quantitative Determination of Cytokine Levels in Prefrontal Cortex of Mice

Firstly, the homogenates of prefrontal cortex were prepared: Tissues one more time were rinsed by ice-cold PBS thoroughly and weighed, then tissues were cut into smaller pieces and homogenized on ice in PBS (w:v = 1:2). The supernatant was obtained by centrifuging the mixtures at 10,000 r.p.m. for 5 min at 4 °C.

Cytokines concentrations in supernatants were assessed by using commercially mouse enzyme-linked immunosorbent assay (ELISA Kit for mice Interleukin IL-1*β*, IL-6 and TNF-*α*, Cloud-Clone Corp., Houston, TX, USA). Before assay, all reagents were brought to room temperature. All samples were tested individually and according to manufacturer’s instruction. The optical density of individual wells was measured by a spectrophotometric microplate reader (BioTek, Elx808, Warszawa, Poland) at a wavelength of 450 nm. The concentration of cytokens in samples were determined by comparing the optical density of the samples to the standard curve. Cytokine concentrations in prefrontal cortex were expressed in picograms per mg protein. Protein concentration was determined according to the method of Bradford [90].

### 4.6. Quantitative Determination of Insulin Level in Prefrontal Cortex of Mice

Insulin concentration in supernatants (obtained after centrifuging the prefrontal cortex homogenates as described above) was assessed by using mouse enzyme-linked immunosorbent assay (ELISA) kit (Mercodia AB, Sweden). The microplate was coated with monoclonal antibodies directed to the insulin. Samples, standards and controls were pipetted into wells. During incubation (37 °C at the time indicated in the procedure) insulin in the samples or standards bound with immobilized anti-insulin antibodies. Then conjugate antibodies were added to the wells (peroxidase-conjugated antibodies) which bind insulin and form something like a sandwich. In the next step, after extensive washing of wells, substrate was added and the plate was incubated at room temperature by 15 min. Substrate was converted to a colored compound by the action of horseradish peroxidase (HRP). There reaction stopped after the addition of a stop solution to give a colorimetric endpoint that is read at a wavelength of 450 nm using a microplate reader (Bio Tek, Poland). The absorbance of the solution was proportional to the amount of insulin in the sample and were read from the standard curve. Insulin concentration in prefrontal cortex were expressed in picograms per mg protein. Protein concentration was determined according to the method of Bradford [94].

### 4.7. Quantitative RT-PCR

#### 4.7.1. Tissue Material Collection and Total RNA Isolation

The tested mice were sacrificed by decapitation and the whole brain was carefully taken out and rinsed in ice-cold saline to remove blood. The prefrontal cerebral cortex and hippocampus were rapidly dissected and used for the study. According to the manufacturer’s instructions, the nucleic acid was extracted from 50 mg of tissue using TRIzol Reagent (Invitrogen, Carlsbad, CA, USA).

#### 4.7.2. Selection of a Reference Gene

The reference genes were selected among hypoxanthine guanine phosphoribosyl transferase (*Hprt*), phosphoglycerate kinase 1 (*Pgk1*) and TATA box binding protein (*Tbp*) based on our preliminary studies, where *Pgk1* and *Tbp* were the most stable reference genes in the cortex and hippocampus. They were not affected by the experimental conditions.

#### 4.7.3. Reverse Transcription and Quantitative Polymerase Chain Reaction

All samples of good quality (OD 260/280 ratios approximately 2.0) were reverse transcribed using random primers and NG dART RT-PCR reagents (EURx, Gdańsk, Poland) as described by the manufacturer. To analyze the correlation of diabetes with the cognitive function, the following primers were used: *Arc*, *Bdnf*, *Egr1* [see Table 1]. The analysis of the genes’ expression levels was performed by real-time PCR method using the 7500 Fast Real-Time PCR System (Applied Biosystems, Foster City, CA, USA) and Fast Probe qPCR Master Mix (2x), plus ROX Solution (EURx, Poland). Briefly, the reaction mixture contained 10 µL of Fast Probe qPCR Master Mix (2x), 9 µL of RNase-free water, ROX Solution (50 nM), and 0.5 µM of gene-specific TaqMan probe (Applied Biosystems, Foster City, CA, USA). The reactions were performed as followed: 95 °C for 3 min, 40 cycles: 95 °C for 10 s and 60 °C for 30 s. The data quality screen based on amplification, Tm and Ct values was performed to remove any outlier data before ΔΔCt calculations and to determine fold change in mRNA levels. The data were presented as a mean RQ ± SEM value (RQ = 2−ΔΔCt).

### 4.8. Statistical Analysis

Differences between two groups were analyzed by unpaired student’s *t*-test. Statistical significance was considered at *p*  <  0.05, *p*  <  0.001 and *p* < 0.0001 and all values are presented as the means  ±  standard error of the mean (SEM). All the statistical analyses were performed using GraphPad Prism software, version 8.0.

## 5. Conclusions

Our studies confirms the existence of a close relationship between hyperglycemia, hyperinsulinemia, and neuroinflammation, and cognitive dysfunction in T2M mouse model. Recorded cognitive impairment in mice with the early stage of diabetes was associated not only with increased levels of cytokines but also decreased *Arc* and *Egr1* mRNA expression level responsible for synaptic plasticity in brain regions associated with learning process and long-term memory formation. The degree of impairment of cognitive functions was proportionate to the decrease in the expression of the marked genes and increase in neuroinflammatory indicators levels. These indicators may be useful to test new forms of treatments of cognitive dysfunction associated not only with diabetes but other diseases manifesting this type of disorders. Moreover, significant changes in *Arc* and *Egr1* memory gene expression in early stage diabetes create opportunities it possible to use them to track the progression of CNS dysfunction and also to differential disease diagnosis running with cognitive impairment.

## Figures and Tables

**Table 1:** The table shows the data on used primers: Gene symbols, assay IDs, gene names, GenBank reference sequence accession numbers and amplicon lengths (bp).

Gene Symbol	Assay ID	Gene Name	RefSeq	Amplicon Length (bp)
*Arc*	AB ID: Mm00479619_g1	activity regulated cytoskeletal-associated protein	NM_018790.3	71
*Bdnf*	AB ID: Mm01334047_m1	brain derived neurotrophic factor	NM_007540.4	105
*Egr1*	AB ID: Mm00656724_m1	early growth response 1	NM_007913.5	182
*Pgk1*	AB ID: Mm01225301_m1	phosphoglycerate kinase 1	NM_008828.3	60
*Tbp*	AB ID: Mm00446974_m1	TATA box binding protein	NM_013684.3	105

AB ID, Applied Biosystems TaqMan Gene Expression Assay ID.

## Data Availability

Not applicable.

## References

[1] WHO Diabetes Statistics Reports. http://www.who.int/mediacentre/factsheets/fs312/en/.

[2] Zimmet P.Z., George K., Alberti M.M. (2016). Epidemiology of Diabetes—Status of a Pandemic and Issues around Metabolic Surgery. Diabetes Care.

[3] Dronavalli S., Duka I., Bakris G.L. (2008). The pathogenesis of diabetic nephropathy. Nat. Clin. Pract. Endocrinol. Metab..

[4] Creager M.A., Lüscher T.F., Cosentino F., Beckman J.A. (2003). Diabetes and vascular disease: Pathophysiology, clinical consequences, and medical therapy: Part I. Circulation.

[5] Veves A., Malik R.A. (2007). Diabetic Neuropathy: Clinical Management (Clinical Diabetes).

[6] Edwards J.L., Vincent A., Cheng T., Feldman E.L. (2008). Diabetic neuropathy: Mechanisms to management. Pharmacol. Ther..

[7] Callaghan B.C., Cheng H.T., Stables C.L., Smith A.L., Feldman E.L. (2012). Diabetic neuropathy: Clinical manifestations and current treatments. Lancet Neurol..

[8] Boulton A.J.M., Vileikyte L., Ragnarson-Tennvall G., Apelqvist J. (2005). The global burden of diabetic foot disease. Lancet.

[9] Luitse M.J.A., Biessels G.J., Rutten E.H.M.L., Kappelle J. (2012). Diabetes, hyperglycaemia, and acute ischaemic stroke. Lancet Neurol..

[10] Duff M., Demidova O., Blackburn S., Shubrook J. (2015). Cutaneous manifestations of diabetes mellitus. Clin. Diabetes.

[11] Moheet A., Mangia S., Seaquist E.R. (2015). Impact of diabetes on cognitive function and brain structure. Ann. N. Y. Acad. Sci..

[12] Papanas N., Ziegler D. (2015). Risk factors and comorbidities in diabetic neuropathy. Rev. Diabet. Stud..

[13] Ceriello A., Hanefeld M., Leiter L. (2004). Postprandial glucose regulation and diabetic complications. Arch. Intern. Med..

[14] Bree A.J., Puente E.C., Daphna-Iken D., Fisher S.J. (2009). Diabetes increases brain damage caused by severe hypoglycemia. Am. J. Physiol. Endocrinol. Metab..

[15] Kanwal R., Akash M.S.H. (2016). Mechanisms of inflammatory responses and development of insulin resistance: How are they interlinked?. J. Biomed. Sci..

[16] Chatterjee S., Peters S.A., Woodward M., Arango S.M., Batty G.D., Beckett N., Beiser A., Borenstein A.R., Crane P.K., Haan M.N. (2016). Type 2 Diabetes as a Risk Factor for Dementia in Women Compared With Men: A Pooled Analysis of 2.3 Million People Comprising More Than 100,000 Cases of Dementia. Diabetes Care.

[17] Cheng G., Huang C., Deng H., Wang H. (2012). Diabetes as a risk factor for dementia and mild cognitive impairment: A meta-analysis of longitudinal studies. J. Intern. Med..

[18] Kandimalla R., Thirumala V., Reddy P.H. (2017). Is Alzheimer’s disease a type 3 diabetes? A critical appraisal. Biochim. Biophys. Acta.

[19] Ma L., Wang J., Li Y. (2015). Insulin resistance and cognitive dysfunction. Clin. Chim. Acta.

[20] McNay E.C., Recknagel A.K. (2011). Brain insulin signaling: A key component of cognitive processes and a potential basis for cognitive impairment in type 2 diabetes. Neurobiol. Learn. Mem..

[21] Perzyńska-Chmiel I., Derkacz M., Schabowski J. (2008). Czy ośrodkowe powikłania cukrzycy mogą przyczynić się do złego wyrównania metabolicznego choroby?. Forum Medycyny Rodzinnej.

[22] Ravona-Springer R., Schnaider-Beeri M. (2011). The association of diabetes and dementia and possible implications for nondiabetic populations. Expert Rev. Neurother..

[23] Ejma M. (2010). Neurologiczne powikłania cukrzycy. Pol. Przegl. Neurol..

[24] Zhao X., Han Q., Lv Y., Sun L., Gang X., Wang G. (2018). Biomarkers for cognitive decline in patients with diabetes mellitus: Evidence from clinical studies. Oncotarget.

[25] Chami B., Steel A.J., De la Monte S.M., Sutherland G.T. (2016). The rise and fall of insulin signaling in Alzheimer’s disease. Metab. Brain Dis..

[26] De la Monte S.M. (2014). Relationships between diabetes and cognitive impairment. Endocrinol. Metab. Clin. N. Am..

[27] Zilliox L.A., Chadrasekaran K., Kwan J.Y., Russell J.W. (2016). Diabetes and cognitive impairment. Curr. Diab. Rep..

[28] Rad S.K., Arya A., Karimian H., Madhavan P., Rizwan F., Koshy S., Prabhu G. (2018). Mechanism involved in insulin resistance via accumulation of β-amyloid and neurofibrillary tangles: Link between type 2 diabetes and Alzheimer’s disease. Drug Des. Dev. Ther..

[29] Jung H.J. (2013). Hyperphosphorylated tau is found in T2DM rat brains Age-dependent increases in tau phosphorylation in the brains of type 2 diabetic rats correlate with a reduced expression of p62. Exp. Neurol..

[30] Gaspar J.M., Babtista F.I., Macedo M.P., Ambrósio A.F. (2016). Inside the diabetic brain: Role of different players involved in cognitive decline. ACS Chem. Neurosci..

[31] Puig K.L., Floden A.M., Adhikari R., Golovko M.Y., Combs C.K. (2012). Amyloid precursor protein and proinflammatory changes are regulated in brain and adipose tissue in a murine model of high fat diet-induced obesity. PLoS ONE.

[32] Marioni R.E., Strachan M.W., Reynolds R.M., Lowe G.D., Mitchell R.J., Fowkes F.G.R., Frier B.M., Lee A.J., Butcher I., Rumley A. (2010). Association Between Raised Inflammatory Markers and Cognitive Decline in Elderly People With Type 2 Diabetes: The Edinburgh Type 2 Diabetes Study. Diabetes.

[33] Gorska-Ciebiada M., Saryusz-Wolska M., Borkowska A., Ciebiada M., Loba J. (2015). Serum levels of inflammatory markers in depressed elderly patients with diabetes and mild cognitive impairment. PLoS ONE.

[34] Shukla V., Shakya A.K., Perez-Pinzon M.A., Dave K.R. (2017). Cerebral ischemic damage in diabetes: An inflammatory perspective. J. Neuroinflam..

[35] Ormstad H., Aass H.C.D., Amthor K.F., Lund-Sørensen N., Sandvik L. (2011). Serum cytokine and glucose levels as predictors of post stroke fatigue in acute ischemic stroke patients. J. Neurol..

[36] Tilg H., Moschen A.R. (2008). Inflammatory mechanisms in the regulation of insulin resistance. Mol. Med..

[37] Evans J.L., Goldfine I.D., Maddux B.A., Grodsky G.M. (2002). Oxidative stress and stress-activated signaling pathways: A unifying hypothesis of type 2 diabetes. Endocrinol. Rev..

[38] Schuh A.F., Rieder C.R., Chaves L.R.M., Roriz-Cruz M. (2011). Mechanisms of brain aging regulation by insulin: Implications for neurodegeneration in late-onset Alzheimer’s disease. ISRN Neurol..

[39] Yang Y., Hu S.H., Zhang J.H., Zhang M.X. (2006). Alzheimer-like hyperphosphorylation of tau in brains of rats with obesity and type 2 diabetes. Prog. Biochem. Biophys..

[40] Daberkow D.P., Riedy M.D., Kesner R.P., Keefe K.A. (2007). Arc mRNA induction in striatal efferent neurons associated with response learning. Eur. J. Neurosci..

[41] Guzowski J.F., Setlow B., Wagner E.K., McGaugh J.L. (2001). Experience-dependent gene expression in the rat hippocampus after spatial learning: A comparison of the immediate-early genes Arc, c-fos, and zif268. J. Neurosci..

[42] Leung H.W., Foo G.W.Q., Van Dongen A.M.J. (2011). Arc regulates transcription of genes for plasticity, excitability and Alzheimer’s Disease. bioRxiv.

[43] Allen J.A., Yadav P.N., Setola V., Farrell M., Roth B.L. (2011). Schizophrenia risk gene *CAV1* is both pro-psychotic and required for atypical antipsychotic drug actions in vivo. Transl. Psychiatry.

[44] Ao H., Liu B., Li H., Lu L. (2019). Egr1 mediates retinal vascular dysfunction in diabetes mellitus via promoting p53 transcription. J. Cell Mol. Med..

[45] Uchino H., Lindvall O., Siesjö B.K., Kokaia Z. (1997). Hyperglycemia and hypercapnia suppress BDNF gene expression in vulnerable regions after transient forebrain ischemia in the rat. J. Cereb. Blood Flow Metab..

[46] Caletti G., Almeida F.B., Agnes G., Nin M.S., Barros H.M.T., Gomez R. (2015). Antidepressant dose of taurine increases mRNA expression of GABAA receptor α2 subunit and BDNF in the hippocampus of diabetic rats. Behav. Brain Res..

[47] Krabbe K.S., Nielsen A.R., Krogh-Madsen R., Plomgaard P., Rasmussen P., Erikstrup C., Fischer C.P., Lindegaard B., Petersen A.M.W., Taudorf S. (2007). Brain-derived neurotrophic factor (BDNF) and type 2 diabetes. Diabetologia.

[48] Guzowski J.F., Lyford G.L., Stevenson G.D., Houston F.P., McGaugh J.L., Worley P.F., Barnes C.A. (2000). Inhibition of Activity-Dependent Arc Protein Expression in the Rat Hippocampus Impairs the Maintenance of Long-Term Potentiation and the Consolidation of Long-Term Memory. J. Neurosci..

[49] Konrad D., Rudich A., Schoenle E.J. (2007). Improved glucose tolerance in mice receiving intraperitoneal transplantation of normal fat tissue. Diabetologia.

[50] Bogush M., Heldt N.A., Persidsky Y. (2017). Blood brain barrier injury in diabetes: Unrecognized effects on brain and cognition. J. NeuroImmune Pharmacol..

[51] Janelidze S., Hertze J., Nägga K., Nilsson K., Nilsson C., Wennström M., van Westen D., Blennow K., Zetterberg H., Hansson O. (2017). Increased blood-brain barrier permeability is associated with dementia and diabetes but not amyloid pathology or APOE genotype. Neurobiol. Aging.

[52] Cattaneo A., Cattane N., Begni V., Pariante C.M., Riva M.A. (2016). The human BDNF gene: Peripheral gene expression and protein levels as biomarkers for psychiatric disorders. Transl. Psychiatry.

[53] Kim B., Feldman E.L. (2015). Insulin resistance as a key link for the increased risk of cognitive impairment in the metabolic syndrome. Exp. Mol. Med..

[54] Leibson C.L., Rocca W.A., Hanson V.A., Cha R., Kokmen E., O’brien P.C., Palumbo P.J. (1997). Risk of dementia among persons with diabetes mellitus: A population-based cohort study. Am. J. Epidemiol..

[55] Arvanitakis Z., Wilson R.S., Bienias J.L., Evans D.A., Bennett D.A. (2004). Diabetes Mellitus and Risk of Alzheimer Disease and Decline in Cognitive Function. Arch. Neurol..

[56] Barrett E.L., Liu Z., Khamaisi M., King G.L., Klein R., Klein B.E.K., Hughes T.M., Craft S., Freedman B.I., Bowden D.W. (2017). Diabetic Microvascular Disease: An Endocrine Society Scientific Statement. J. Clin. Endocrinol. Metab..

[57] Nakamura J., Kato K., Hamada Y., Nakayama M., Chaya S., Nakashima E., Naruse K., Kasuya Y., Mizubayashi R., Miwa K. (1999). A protein kinase C-beta-selective inhibitor ameliorates neural dysfunction in streptozotocin-induced diabetic rats. Diabetes.

[58] Takeda S., Sato N., Uchio-Yamada K., Sawada K., Kunieda T., Takeuchi D., Kurinami H., Shinohara M., Rakugi H., Morishita R. (2010). Diabetes accelerated memory dysfunction via cerebrovascular inflammation and Aβ deposition in Alzheimer mouse model with diabetes. Proc. Natl. Acad. Sci. USA.

[59] Stevens M.J., Dananberg J., Feldman E.L., Lattimer S.A., Kamijo M., Thomas T.P., Shindo H., Sima A.A., Greene D.A. (1994). The linked roles of nitric oxide, aldose reductase and, (Na+,K+)-ATPase in the slowing of nerve conduction in the streptozotocin diabetic rat. J. Clin. Investig..

[60] Gispen W.H., Biessels G.J. (2000). Cognition and synaptic plasticity in diabetes mellitus. Trends Neurosci..

[61] Zhao W., Chen H., Xu H., Moore E., Meiri N., Quon M.J., Alkon D.L. (1999). Brain insulin receptors and spatial memory. Correlated changes in gene expression, tyrosine phosphorylation, and signaling molecules in the hippocampus of water maze trained rats. J. Biol. Chem..

[62] Whitmer R.A. (2007). Type 2 diabetes and risk of cognitive impairment and dementia. Curr. Neurol. Neurosci. Rep..

[63] Sladek R., Rocheleau G., Rung J., Dina C., Shen L., Serre D., Boutin P., Vincent D.R., Belisle A., Hadjadj S. (2007). A genome-wide association study identifies novel risk loci for type 2 diabetes. Nat. Cell Biol..

[64] Qiu W., Folstein M. (2006). Insulin, insulin-degrading enzyme and amyloid-β peptide in Alzheimers disease: Review and hypothesis. Neurobiol. Aging.

[65] Tang W.J. (2016). Targeting insulin-degrading enzyme to treat type 2 diabetes. Trends Endocrinol. Metab..

[66] Farris W., Mansourian S., Chang Y., Lindsley L., Eckman E.A., Frosch M.P., Eckman C.B., Tanzi R.E., Selkoe D.J., Guenette S. (2003). Insulin-degrading enzyme regulates the levels of insulin, amyloid beta-protein, and the beta-amyloid precursor protein intracellular domain in vivo. Proc. Natl. Acad. Sci. USA.

[67] Lambert G.W., Schlaich M.P., Esler M.D. (2007). Brain derived neurotrophic factor (BDNF) release from the human brain in patients with type 2 diabetes--possible influence of venous anatomy and comorbid major depressive disorder. Diabetologia.

[68] Kauer-Sant’Anna M., Kapczinski F., Andreazza A.C., Bond D.J., Lam R.W., Young L.T., Yatham L.N. (2008). Brain-derived neurotrophic factor and inflammatory markers in patients with early- vs. late-stage bipolar disorder. Int. J. Neuropsychopharmacol..

[69] Skundric D.S., Lisak R.P. (2003). Role of neuropoietic cytokines in development and progression of diabetic polyneuropathy: From glucose metabolism to neurodegeneration. J. Diab. Res..

[70] Mushtaq G., Khan J.A., Kumosani A., Kamal M.A. (2015). Alzheimer’s disease and type 2 diabetes via chronic inflammatory mechanisms. Saudi J. Biol. Sci..

[71] Turrin N.P., Rivest S. (2006). Tumor necrosis factor α but not interleukin 1β mediates neuroprotection in response to acute nitric oxide excitotoxicity. J. Neurosci..

[72] Sochocka M., Diniz B.S., Leszek J. (2017). Inflammatory Response in the CNS: Friend or Foe?. Mol. Neurobiol..

[73] Khandelwal P.J., Herman A.M., Moussa C.E.-H. (2011). Inflammation in the early stages of neuro-degenerative pathology. J. Neuroimmunol..

[74] Franceschi C., Campisi J. (2014). Chronic inflammation (inflammaging) and its potential contribution to age-associated diseases. J. Gerontol. A Biol. Sci. Med. Sci..

[75] Raison C.L., Dantzer R., Kelley K.W., Lawson M.A., Woolwine B.J., Vogt G., Spivey J.R., Saito K., Miller A.H. (2010). CSF concentrations of brain tryptophan and kynureninesduring immune stimulation with IFN-alpha: Relationship to CNS immune responses anddepression. Mol. Psychiatry.

[76] Miller A.H., Haroon E., Raison C.L., Felger J.C. (2013). Cytokine Targets in the Brain: Impact on Neurotransmitters and Neurocircuits. Depress. Anxiety.

[77] Beurel E., Jope R.S. (2006). The paradoxical pro- and anti-apoptotic actions of GSK3 in the intrinsic and extrinsic apoptosis signaling pathways. Prog. Neurobiol..

[78] Datusalia A.K., Sharma S.S. (2014). Amelioration of Diabetes-induced Cognitive Deficits by GSK-3β Inhibition is Attributed to Modulation of Neurotransmitters and Neuroinflammation. Mol. Neurobiol..

[79] Plath N., Ohana O., Dammermann B., Errington M.L., Schmitz D., Gross C., Mao X., Engelsberg A., Mahlke C., Welzl H. (2006). Arc/Arg3.1 Is Essential for the Consolidation of Synaptic Plasticity and Memories. Neuron.

[80] Passaro A., Nora E.D., Morieri M.L., Soavi C., Sanz J.M., Zurlo A., Fellin R., Zuliani G. (2015). Brain-Derived Neurotrophic Factor Plasma Levels: Relationship With Dementia and Diabetes in the Elderly Population. J. Gerontol. Ser. A Biol. Sci. Med. Sci..

[81] Shepherd J.D., Bear M.F. (2011). New views of Arc, a master regulator of synaptic plasticity. Nat. Neurosci..

[82] Gallo F.T., Katche C., Morici J.F., Medina J.H., Weisstaub N.V. (2018). Immediate Early Genes, Memory and Psychiatric Disorders: Focus on c-Fos, Egr1 and Arc. Front. Behav. Neurosci..

[83] Duclot F., Kabba M. (2017). The Role of Early Growth Response 1 (EGR1) in Brain Plasticity and Neuropsychiatric Disorders. Front. Behav. Neurosci..

[84] Hennigan A., Trotter C., Kelly A.M. (2007). Lipopolysaccharide impairs long-term potentiation and recognition memory and increases p75NTR expression in the rat dentate gyrus. Brain Res..

[85] Blalock E.M., Chen K.-C., Sharrow K., Herman J.P., Porter N.M., Foster T.C., Landfield P.W. (2003). Gene microarrays in hippocampal aging: Statistical profiling identifies novel processes correlated with cognitive impairment. J. Neurosci..

[86] Minatohara K., Akiyoshi M., Okuno H. (2016). Role of Immediate-Early Genes in Synaptic Plasticity and Neuronal Ensembles Underlying the Memory Trace. Front. Mol. Neurosci..

[87] Penrod R.D., Kumar J., Smith L.N., McCalley D., Nentwig T.B., Hughes B.W., Barry G.M., Glover K., Taniguchi M., Cowan C.W. (2019). Activity-regulated cytoskeleton-associated protein (Arc/Arg3.1) regulates anxiety- and novelty-related behaviors. Genes Brain Behav..

[88] Naert G., Rivest S. (2012). Age-related changes in synaptic markers and monocyte subsets link the cognitive decline of APP(Swe)/PS1 mice. Front. Cell Neurosci..

[89] Koldamova R., Schug J., Lefterova M., Cronican A.A., Fitz N.F., Davenport F.A., Carter A., Castranio E.L., Lefterov I. (2014). Genome-wide approaches reveal EGR1-controlled regulatory networks associated with neurodegeneration. Neurobiol. Dis..

[90] Leu S.-Y., Kuo L.-H., Weng W.-T., Lien I.-C., Yang C.-C., Hsieh T.-T., Cheng Y.-N., Chien P.-H., Ho L.-C., Chen S.-H. (2020). Loss of EGR-1 uncouples compensatory responses of pancreatic β cells. Theranostics.

[91] Hall C.S., Ballachey E.L. (1932). A study of the rat’s behavior in a field: A contribution to method in comparative psychology. University of California Publications in Psychology.

[92] Venault P., Chapouthier G., de Carvalho L.P., Simiand J., Morre M., Dodd R.H., Rossier J. (1986). Benzodiazepine impairs and beta-carboline enhances performance in learning and memory tasks. Nature.

[93] Ennaceur A., Delacour J. (1988). A new one-trial test for neurobiological studies of memory in rats. Behav. Brain Res..

[94] Bradford M.M. (1976). A rapid sensitive method for the quantification of microgram quantities of protein utilising the principle of protein-Dye Binding. Anal. Biochem..

